# The Janus‐faced effects of COVID‐19 perceptions on family healthy eating behavior: Parent’s negative experience as a mediator and gender as a moderator

**DOI:** 10.1111/sjop.12742

**Published:** 2021-05-31

**Authors:** Ali B. Mahmoud, Dieu Hack‐Polay, Leonora Fuxman, Maria Nicoletti

**Affiliations:** ^1^ St. John's University New York USA; ^2^ University of Lincoln Lincoln UK; ^3^ NHS Scotland Glasgow UK

**Keywords:** Attitudes, COVID‐19 perceptions, food, gender, healthy eating behavior, negative emotions, pandemic time, parenting

## Abstract

This research examines the effects of COVID‐19 perceptions and negative experiences during the pandemic time on parental healthy eating behavior and whether these relationships interact with a parent’s gender. We ran a survey of parents who had at least one child aged 3 to 17 years old living in the United Kingdom. We received 384 valid responses, which were analysed via a variance‐based structural equation modeling approach to test our hypotheses. The results revealed that COVID‐19 perceptions effects were Janus‐faced. While they indirectly and negatively impact healthy eating behavior mediated by triggering negative experiences during the pandemic, COVID‐19 perceptions, however, directly get parents, especially fathers, more engaged into healthy eating behavior – making COVID‐19 perceptions total effects positive on healthy eating behavior. This explorative model is novel in the sense that it is the first of its kind to cast light on how parental healthy eating behavior can be shaped in pandemic time. The research is particularly timely due to the uncertain times in which the research is situated, that is, the worldwide pandemic (also termed COVID‐19); the paper highlights how family eating practices can undergo dramatic shifts during acute crises.

## Introduction

The COVID‐19 pandemic has shaken all aspects of life, economics, politics, family relationships, and social life more broadly (Chakraborty & Maity, 2020). Eating habits within families have not escaped this dynamic of change occasioned by the pandemic (Di Renzo *et al*., 2020; Mahmoud, Hack‐Polay, Fuxman, Naquiallah & Grigoriou, 2020), which can affect children’s dietary intake as it is dependent on their parents’ (e.g., Tang, Bu & Dong, 2020). Several factors condition food choices and eating practices, for example, economics, culture and the social networks that one frequents, etc. (Arnold & Sobal, 2000; Kabir, Miah & Islam, 2018; Mahmoud & Grigoriou, 2019; Mahmoud, Hack‐Polay, Fuxman, Naquiallah *et al*., 2020; Snoek, Van Strien, Janssens & Engels, 2007). Though these factors have attracted significant coverage in the social and medical sciences, the investigation of the factors affecting eating habits and food choices during times of pandemic has not attracted sufficient coverage. The emergence of COVID‐19 has started to highlight the criticality of such research. Mahmoud, Hack‐Polay, Fuxman, Naquiallah *et al*. (2020) alluded to the fact that families with children with allergies, for instance, may show some propensity to source whatever food is available as due to the restrictions, such families are not always able to be at the front of the queues, particularly when the population rush into panic‐buying (Athas, 2019; Chooniedass, Temple, Martin & Becker, 2018; Radcliffe, 2018).

In the past 20 years or so, economic and social dynamics significantly altered how families approached food choices and developed eating habits (Mahmoud, Hack‐Polay, Fuxman, Naquiallah *et al*., 2020; Meza *et al*., 2020). The popularization of fast food and ready‐made meals, as well as their low cost, enticed many families to shift from healthy eating to increasingly embracing the new easy to source food and junk food (Chan, 2011; Chang, Hillier & Mehta, 2009; Mahmoud & Grigoriou, 2019; Mahmoud, Hack‐Polay, Fuxman, Naquiallah *et al*., 2020; Taillie, 2018). The ongoing pandemic has come to add to the socio‐economic determinants of unhealthy food choices as a new and critical datum; unhealthy food is often referred to as food with high levels of sodium, fat and sugar (Mahmoud, Hack‐Polay, Fuxman, Naquiallah *et al*., 2020). These changes have caused children’s diets to be increasingly imbalanced. Accordingly, children’s menus now contain more calories, fat, and sodium than the nutritional proportion advised for their age group (Mahmoud & Grigoriou, 2019; Mahmoud, Hack‐Polay, Fuxman, Naquiallah *et al*., 2020; Nørgaard & Brunsø, 2011). The resulting change in children’s diets has engendered apprehensions about the growing trend in childhood issues such as obesity (Lappan *et al*., 2020). If these issues were persistent during times with relative normality, “the new normal” under COVID‐19 appears to be exacerbating eating behavior in families as they increasingly get priced out of “fresh food” and confined the consumption of food rich in fat and sugar (Minnesota Department of Health, 2020; Vandinther, 2020), which they can store for longer.

It is, therefore, clear that substantial research is required to identify the impact of COVID‐19 on food choices made by families, particularly those with young children, if targeted policy frameworks must be devised to reduce the impact of the pandemic on children’s health and poverty (UNICEF, 2020; Van Lancker & Parolin, 2020). Delay in such research will confine over 150 million children to poverty, leading to serious health consequences and significant infantile mortality (UNICEF, 2020). Also, a recent survey by the Royal College of Paediatrics and Child Health (Mundasad, 2020) shows that some parts of Great Britain have witnessed a three‐ to a four‐fold rise in cases of eating disorder in 2020 compared to 2019. The survey also warns parents that they need to be on the look‐out for any signs of behavioral eating disorders among children and young people (Mundasad, 2020), offering a rationale for studying the effects of COVID‐19 on family eating behavior.

Our study, thus, endeavors to be one of the first critical studies in this context. Therefore, the study aims to examine the effects of COVID‐19 perceptions and negative experiences on parental healthy eating behavior in the United Kingdom amidst the coronavirus pandemic’s outbreak. We specifically examine the degree to which these relationships interact with parents’ gender, since gender differences are still significant and prevalent in society, especially in the context of home and the family (Bove, Sobal & Rauschenbach, 2003; Singh & Mukherjee, 2018) as well as the perceptions of crisis and proneness to stressful experiences (Fehm, Pelissolo, Furmark & Wittchen, 2005). That will be achieved through developing and testing a structural path model that links COVID‐19 perceptions and negative experience effects on family healthy eating. Additionally, we examine the moderating role of parents’ gender in the variance in healthy eating behavior *hypothetically* triggered by parents’ COVID‐19 perceptions and negative experience using a variance‐based (or partial least square) structural equation modeling approach. Our study is the first to examine the complex relationships between variables under consideration where COVID‐19 perceptions are assessed using an independent measure (Mahmoud, Grigoriou, Fuxman, Reisel, Hack‐Polay *et al*., 2020). The COVID‐19 study field is new, and we have not encountered a similar study exploring the relationships between the variables that we have considered, thus adding to the literature.

## Theoretical foundations and hypotheses

### Parents’ COVID‐19 perceptions and familial healthy eating behavior

The COVID‐19 pandemic has affected most of the world, causing lockdowns in many countries, with varying degree of strictness but with the common goal to limit virus transmissions by restricting social interactions, thus “social distancing” and “sheltering in place” policies to encourage people to stay away from each other and remain home. With many individuals shifting to remote work from home and with many traditional food choices such as restaurants being unavailable, individual’s food choices and consumption patterns have been greatly impacted during the lockdowns and afterwards (see Jia, Liu, Xie *et al*., 2021; Marty, de Lauzon‐Guillain, Labesse & Nicklaus, 2021).

COVID‐19 perceptions defined by Mahmoud, Grigoriou, Fuxman, Reisel, Hack‐Polay *et al*. (2020) as the perceived probability of discomfort and/or worry, during COVID‐19 pandemic, concerning the pandemic adverse health, economic and social ramifications articulated as disruptions to the people’s pre‐pandemic everyday life – lead to redefining of the everyday life to the “new normal.” Many countries reported food shortages at the start of the pandemic primarily associated with lockdowns and interruptions in the supply chain, which research indicates lead to significant alterations of food consumption patterns and consequently to nutritional changes for most categories of populations, including kids, young people, and adults (see Benker, 2021; Brown, Opitz, Peebles, Sharpe, Duffy & Newman, 2021; Robinson, Boyland, Chisholm *et al*., 2021; Shen, Long, Shih & Ludy, 2020; Valentin, Sylvain, Oulmann *et al*., 2020; Vazquez‐Vazquez, Dib, Rougeaux, Wells & Fewtrell, 2021). While food shortages and associated behavioral changes in food choices were mostly only temporary, the new “pandemic lifestyle” has gained its permanence during much of 2020 and now into the year 2021. The manifestation of psychological, emotional and cognitive distress over the continued pandemic state has been linked to alterations in food choices (e.g., Marty *et al*., 2021; Mattioli, Ballerini Puviani, Nasi & Farinetti, 2020; Poelman, Gillebaart, Schlinkert *et al*., 2021; Shen *et al*., 2020) with some evidence suggesting pandemic has helped to boost health consideration choices for some, while for others it contributed to unhealthy mood‐related consumption as a means to reduce pandemic‐related stress. In connection with this, the Theory of Planned Behavior (TPB) (Ajzen, 1991) is relevant. TPB contends that the plan for a particular behavior is conditioned by three factors: individual attitude, social influence and self‐efficacy. This suggests that the individual’s negative or positive view of healthy food choices, together with societal pressure and the ease of performing a given behavior, will act as key determinants of family food choices. Previous studies (Steptoe, Pollard & Wardle, 1995) suggest that several possible factors impact food choice decisions, including cost, convenience and personality, etc. These variables are relevant to crises. Thus, in this study, in particular, we focus on parental food decisions as they impact children’s eating patterns. Thus, our desire to explore if and how parents’ attitudes and behavior towards familial healthy eating habits are being affected by their COVID‐19 perceptions. Hence, we formulate our first exploratory hypothesis:

Hypothesis 1 Parents’ COVID‐19 perceptions will impact familial healthy eating behavior.

### Parental negative emotions/experience and familial healthy eating behavior

Prolonged exposure to the COVID‐19 pandemic puts significant stress on families by increasing parental worry and anxiety about the uncertain future. While the social distancing lockdowns altered familial behavior at home (e.g., no access to daycare and/or in‐person school) and caused lifestyle changes (e.g., no access to kids sports/recreation), mental health has been hit hard by the pandemic’s social isolation consequence (see Daly, Sutin & Robinson, 2020; O’Connor *et al*., 2020). Neither parents nor children are immune to mental and behavioral health issues. For example, an early US national survey conducted in June of 2020 (Patrick, Henkhaus, Zickafoose *et al*., 2020) indicates that 27% of parents reported worsening of their mental health while 14% reported worsening of their children’s behavioral state, with approximately 1 in 10 families experiencing concurrent worsening for parents and children. As suggested by the Theory of Planned Behavior, these social pressures would lead families to assess the desirability and adoption of particular eating behavior, that is, unhealthy eating or healthy eating (Diener, Wirtz, Tov *et al*., 2010; Steptoe *et al*., 1995).

While previous research is consistent in suggesting a decline in adult physiological well‐being during crises caused by either lengthy economic downturns or natural disasters (see Ananat, Gassman‐Pines, Francis & Gibson‐Davis, 2017; Gassman‐Pines, Ananat & Fitz‐Henley, 2020), there is no research to help us understand the complexities of responses to the COVID‐19 pandemic from the viewpoint of one of the more vulnerable population groups such as family where parental mental well‐being is directly linked to children’s psychological well‐being (Smith, 2004). Given our understanding of COVID‐19 impact on emotional and mental well‐being, we anticipate the connection between the intensity of parental COVID‐19 perceptions and negative emotions as follows:

Hypothesis 2 More intense COVID‐19 perceptions will trigger more negative emotions/experience among parents

Existing early research indicates that the coronavirus has impacted parents and their children’s physical and emotional well‐being in several negative ways. For example, in relation to nutrition and food consumption, increased food insecurity and amended access to public food assistance (Patrick *et al*., 2020) coupled with exceedingly sedentary lifestyles due to stay‐in‐place policies (Robinson *et al*., 2021) and strained mental state (O’Connor *et al*., 2020) all have potential to manifest in health‐damaging familial food consumption behavior.

While an individual’s eating behavior is often attributed to habits that have a tendency to be formed within a similar context and repeat over time, the disruption to the habitual food selection decision process brought on by the crisis‐like global coronavirus pandemic has been linked to underlying changes in motives that drive individuals to particular food selections (Marty *et al*., 2021). The actual fabric behind such motives includes social, cultural, political, and contextual factors and the more conventional factors of nutritional values and visual/sensual appeal associated with the food selection (Diener *et al*., 2010; Kittler, Sucher & Nelms, 2017).

Negative emotions such as stress, anxiety, depression have all been linked to undesirable eating consequences (Shen *et al*., 2020). Emotional eating, which occurs when eating without following an internal hunger cues but rather in response to negative emotions such as, for example, perceived stress, has been linked to the development of disagreeable health factors including obesity, stress, depression and an increase in a variety of undesirable nutritional intakes such as sugars, fats, sodium (Shen *et al*., 2020). Thus, Diener *et al*., (2010) argue that the psychological capital at the disposal of an individual is critical in food behavior adoption.

Given the theoretical and empirical evidence, we formulate our following hypothesis:

Hypothesis 3 Parents’ negative emotions/experience will impact healthy eating behavior

### Parent gender and familial healthy eating behavior

Research on gender variations in responses to stress stimulus suggests that while both genders are at risk with adverse physiological and psychological outcomes, women are more prone to developing depression and anxiety in response to stress compared to men who are more prone to developing physiological outcomes, for example, high blood pressure (Chaplin, Hong, Bergquist & Sinha, 2008). Within the context of familial food selection, it is worth noting that prior studies indicate a female propensity to be more influenced by emotional eating bearing the bulk of negative consequences such as weight gain compared to male counterparts (Bennett, Greene & Schwartz‐Barcott, 2013; Snoek *et al*., 2007). The pandemic state’s sustained gravity continues to complicate the balance of COVID‐19 perceptions factors impacting parental emotions and creating sustained negative experiences that end up impacting healthy eating behavior. We expect parental gender to be a moderating factor. This assumption is founded on emerging literature supporting gender differences in psychological capital (Chawla & Sharma, 2019; Rani & Chaturvedula, 2018).

**Fig. 1:**
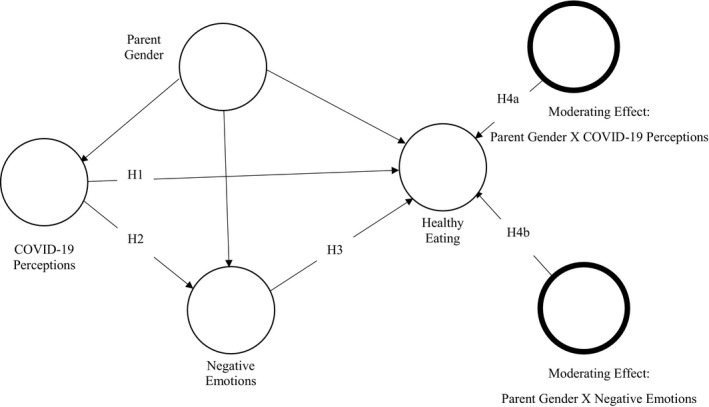
Hypothesised model.

While parental feeding practices and children’s eating patterns have been described in the literature, most studies are based on mothers’ attitudes as primary caregivers involved in food‐related decisions and meals preparation (Lipowska *et al*., 2018). Mahmoud, Hack‐Polay, Fuxman, Naquiallah *et al*. (2020) mention, in their study of allergy‐prone children, the difference in the way mothers and fathers approach the child’s food selection process when maintaining a healthy food balance is critical to child safety. Parental socio‐demographic attributes, lifestyle, education, and nutritional awareness have been considered factors that pave a deep connection to children’s food choices and lifestyles later in life (Nazzaro, Lerro & Marotta, 2018). Based on these theories, we hypothesize Fig. 1:

Hypothesis 4a Parents’ gender will moderate the effect of COVID‐19 perceptions on their familial healthy eating behavior

Hypothesis 4b Parents’ gender will moderate the effect of parents’ negative emotions/experience on their familial healthy eating behavior

## Methods

Our study took place in the United Kingdom between April 2020 and December 2020. Given the extreme pandemic context, we utilized a convenience sampling through which parents of children aged 3–17 in the UK were approached via social networks. A total of 1,000 potential participants were contacted. The entire sampling process yielded 384 usable responses, and we followed a quantitative approach to analyzing the data. This consisted mainly of partial least square structural equation modeling (PLS‐SEM) to test the proposed structural model via SmartPLS 3 (Ringle, Wende & Becker, 2015). PLS‐SEM has obtained more academic favorability when testing predictive models (Hair, Ringle & Sarstedt, 2014; Mahmoud, Reisel, Fuxman & Mohr, 2021). Most data are expected to violate the criterion of multivariate normality (Mahmoud, Hack‐Polay, Fuxman, Massetti *et al*., 2020; Mahmoud *et al*., 2021); therefore, the PLS‐SEM method has earned substantial recognition for empirical investigations where data are susceptible to non‐normality issues (Hair, Gassman‐Pines, Francis & Gibson‐Davis, 2017).

We utilised the work of Mahmoud, Grigoriou, Fuxman, Reisel, Hack‐Polay *et al*. (2020) to measure COVID‐19 perceptions, Diener *et al*., (2010) to measure negative experience and Steptoe *et al*., (1995) to measure healthy eating behavior (see Appendix Table A1). All of the attitudinal measuring items were assessed on a five‐point Likert scale. Table 1 reports heterotrait‐monotrait (HTMT) ratios less than 0.9 confirming that the measures utilized satisfied the discriminant validity criterion (Hair, Risher, Sarstedt & Ringle, 2019). Table 2 indicates that all the constructs had average variance extracted (AVEs) higher than 0.5, composite reliability scores (CRs) between 0.874 and 0.897 satisfying the convergent validity and reliability criteria for all measures (Hair *et al*., 2019; Hair, Sarstedt, Ringle & Gudergan, 2018). Also, Table 3 shows that all variance inflation factor (VIFs) values were less than 5 offering evidence that there were no collinearity issues (Hair *et al*., 2018) Fig. 2.

**Table 1:** Discriminant validity test (HTMT)

	COVID‐19 Perceptions	Healthy eating
Healthy eating	0.241	
Negative emotions	0.728	0.125

**Table 2:** Outer loadings, VIFs, construct reliability and validity

Item	COVID‐19 Perceptions	Healthy Eating	Negative Emotions	VIF
Afraid			0.724	1.697
Angry			0.603	1.461
Bad			0.701	2.819
Negative			0.897	2.896
Sad			0.612	2.11
Unpleasant			0.836	2.183
cov01	0.769			1.293
cov02	0.859			1.403
cov03	0.952			1.098
hfch02		0.734		3.612
hfch03		0.771		3.055
hfch04		0.793		1.564
hfch05		0.743		3.232
hfch06		0.69		1.728
hfch01		0.698		1.596
Cronbach's Alpha	0.889	0.878	0.874	
rho_A	0.906	0.88	0.888	
Composite reliability	0.897	0.878	0.874	
Average variance extracted (AVE)	0.746	0.546	0.543	

**Table 3:** Inner VIFs values

	COVID‐19 Perceptions	Healthy eating	Negative emotions
COVID‐19 perceptions		1.582	
Negative emotions		1.582	
Healthy eating	1.006		
Negative emotions	1.006		
COVID‐19 perceptions			1.03
Healthy eating			1.03

**Fig. 2:**
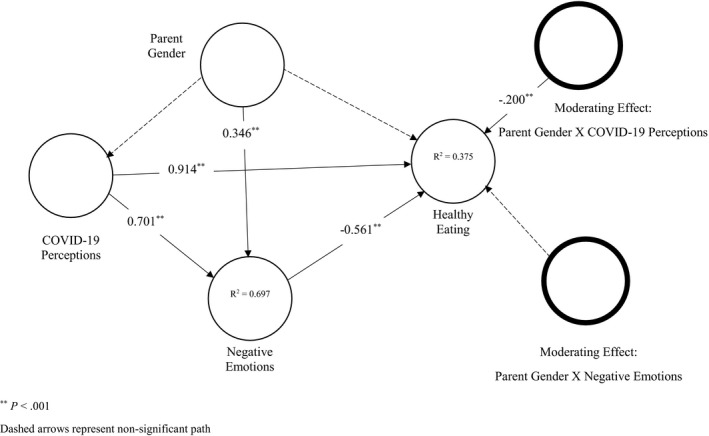
Alternate model.

We conducted a path assessment to test the hypotheses that included reporting standardized betas (β) for direct effects, unstandardized betas (B) for indirect effects and their *t*‐values with consistent‐PLS bootstrapping at 5,000 samples (Preacher & Hayes, 2008). Moreover, Cohen’s f^2^ was used to assess effect sizes, where scores ≥ 0.02, f^2^ ≥ 0.15 and f^2^ ≥ 0.35 express small, medium and large effect sizes, respectively (Cohen, 1988). Besides, Q^2^ was used to assess predictive relevance and the standard root mean square residual (SRMR) was utilized to evaluate the model fit to the data (Henseler, Dijkstra, Sarstedt *et al*., 2014).

We conducted common‐method bias (CMB) tests, which is necessary when using cognitive, self‐reported measures from a single study (Podsakoff, MacKenzie, Lee & Podsakoff, 2003). All the inner variance inflation factors (VIFs) returned scores of less than 3.3 (See Table 3). Subsequently, no multicollinearity or CMB concerns were found (Kock, 2015).

## Results

### Sample description

We computed the arithmetic means and standard deviations for the latent variables utilizing SPSS version 26, the sample majority was found to be mothers (78%), with no food allergy (90%), educated to a Higher National Certificate/A‐level, National Diploma/GCSE or lower (50%), aged between 30 and 40 years (65%), and with children in their middle childhood (41%) with no food allergy (92%). Table A2 shows the descriptive statistics of COVID‐19 perceptions, negative emotions and healthy eating categorized according to the parent’s gender. It suggests that mothers experience more intense COVID‐19 perceptions and negative emotions than fathers.

### Path analysis

Given the reflective nature of the latent variables in our model (Mahmoud *et al*., 2021), we performed Consistent‐PLS Algorithm, followed by Consistent PLS Bootstrapping run at 5,000 sub‐samples (Preacher & Hayes, 2008) in order to analyze the hypothesized path model.

COVID‐19 perceptions are found to positively predict negative emotions (β = 0.701, *p* < 0.001, f^2^ > 0.35) and healthy eating (β = 0.914, *p* < 0.001, f^2^ > 0.35). Besides, we find that negative emotions negatively predict healthy eating (β = −0.561, *p* < 0.001, f^2^ > 0.15). Hence, we conclude that H1, H2 and H3 are supported (See Table 4). The path linking parent’s gender to negative emotions is found to be significantly positive (β = 0.346, *p* < 0.001, f^2^ > 0.35) implying that mothers are more likely to experience negative emotions during the current pandemic than fathers. While parents’ gender effects on COVID‐19 perceptions (β = 0.018, *p* > 0.05, f^2^ < 0.02) and healthy eating (β = 0.096, *p* < 0.05, f^2^ < 0.02) are found to be non‐significant or not large enough, it is found, however, to negatively and significantly interact with/moderate COVID‐19 perceptions effects on healthy eating (β = −0.200, *p* < 0.001, f^2^ > 0.02) offering evidence that mothers are less likely to engage in healthy eating as a response to COVID‐19 perceptions. The moderating effect: parent gender × negative emotions, however, is found non‐significant (β = −0.002, *p* > 0.05, f^2^ < 0.02). Therefore, we judge H4a as supported and H4b as unsupported.

**Table 4:** Hypotheses testing– Direct paths

Hypothesis	Path	β	*t*	f^2^	Decision
H1	COVID‐19 Perceptions ‐> Healthy Eating	0.914	11.119**	> 0.35	Supported
H2	COVID‐19 Perceptions ‐> Negative Emotions	0.701	24.442**	> 0.35	Supported
H3	Negative Emotions ‐> Healthy Eating	−0.561	5.511**	> 0.15	Supported
Additional	Parent Gender ‐> Healthy Eating	0.096	2.430*	< 0.02	Rejected
Additional	Parent Gender ‐> COVID‐19 Perceptions	0.018	1.082*^NS^*	< 0.02	Rejected
Additional	Parent Gender ‐> Negative Emotions	0.346	14.483**	> 0.35	Supported
H4a	Moderating Effect: Parent Gender X COVID‐19 Perceptions ‐> Healthy Eating	−0.200	4.506**	> 0.02	Supported
H4b	Moderating Effect: Parent Gender X Negative Emotions ‐> Healthy Eating	−0.002	0.023*^NS^*	< 0.02	Rejected

Notes

*NS = *Non‐significant.

** *p* < 0.001;

* *p* < 0.05.

Table 5 shows the assessment of the indirect effects. Our statistics suggest that negative emotions fully mediate the relationship between gender and healthy eating (B = −0.194, STDV = 0.039, *P* < 0.001). This means mothers are less likely to engage in healthy eating practices because of the negative emotions/experience than fathers. Also, negative emotions are found to partially mediate the relationship between COVID‐19 perceptions and healthy eating (B = −0.394, STDV = 0.074, *p* < 0.001), implying that COVID‐19 perceptions lead to less engagement in healthy eating because of the negative emotions triggered during the pandemic. Since this result contradicts the positive direct effect of COVID‐19 perceptions on healthy eating, we look at the total effect of COVID‐19 perceptions on healthy eating, which shows that the effects remain positive (B = 0.519, STDV = 0.049, *p* < 0.001) despite being *attacked* by the negative emotions and the interaction with parents’ gender.

**Table 5:** Indirect paths

Path	B	STDEV	*t*
COVID‐19 Perceptions ‐> Negative Emotions ‐> Healthy Eating	−0.394	0.074	5.41**
Parent Gender ‐> Negative Emotions ‐> Healthy Eating	−0.194	0.039	5.031**

** *p* < 0.001.

Ultimately, with SRMR equal to 0.038 < 0.08, we decide that our alternate model is an excellent fit for our data (Hu & Bentler, 1999). Table 6 shows that the Q^2^ values of all the predictors larger than 0, which suggests sufficient predictive relevance. Also, R^2^ values for negative emotions (0.697) and healthy eating (0.375) were all higher than zero, suggesting that our model, according to Cohen (1988), possesses substantial predictive accuracy.

**Table 6:** Predictive relevance (Q^2^)

	SSO	SSE	Q² (=1‐SSE/SSO)
Healthy eating	1152.00	890.433	0.227
Negative emotions	2304.00	1329.80	0.423

### Discussion and conclusion

The coronavirus pandemic or COVID‐19 has been communicated in the contemporary literature (and anecdotal reports) as a traumatizing event that split humankind history between two distinct eras, that is, pre‐and post‐pandemic – establishing the “new normal.” The “new normal” (or the state following the pandemic crisis) has witnessed drastic changes in people’s attitudes and behavior across all aspects of life, including family eating behavior. Therefore, the current study was conducted in response to calls by scholars and policymakers to engage in empirical investigations that would advance the publics’ evidence‐based understanding of the effects of COVID‐19 and associated experiences on family eating behavior’s healthiness.

In reviewing the literature, no data were found to analyse the relationships among parents’ gender, COVID‐19 perceptions, negative experience, and healthy eating behavior. To our best knowledge, employing an independent measure of COVID‐19 perceptions was not utilized in previous research on family eating practices. Therefore, our study has addressed this research gap. It has hypothesized a structural model and tested it using a PLS‐SEM method.

The results of this analysis supported H1, H2, H3 and H4a and rejected H4b. Also, only one additional path (parent gender → negative emotions/experience) was found significant and meaningful. All of the indirect effects explored were found significant. Although triggering negative experience during the vicious pandemic and those negative emotions lead to less engagement in family healthy eating, surprisingly, COVID‐19 perceptions were found to exert a positive total effect over familial healthy eating, especially among fathers. In other words, whilst COVID‐19 perceptions have indirectly led to lower parental engagement levels in family healthy eating behavior through prompting negative emotions, COVID‐19 perceptions have pushed parents into more adoption of healthy eating behavior, especially among fathers. Adopting healthier eating behavior amidst the spread of diseases is a likely response by parents to support the family members’ immune system. For instance, a recent study in Italy (Di Renzo *et al*., 2020) found that people exhibited more interest in Mediterranean food during the lockdown and showed more adherence to healthy diets rich in minerals and vitamins that could enhance the functionality of the immune system. Another study (Ben Hassen, El Bilali & Allahyari, 2020) in a Middle Eastern context observed a greater tendency towards healthier diets post the outbreak of COVID‐19.

Additionally, the contemporary discourse on food habits has highlighted how people have changed their way of shopping and preparing food. For example, greater interest in reading labels, learning about where foods come from, understanding what ingredients are in the foods, and which foods they should avoid have been noted among shoppers (Lempert, 2020). In the UK, a new report, carried out by the food standards agency (FSA) and Ipsos Mori, found that many Britons, during the pandemic, observed positive changes to household food behavior that they wished to continue as lockdown lifted or eased. Examples of those changes were increased food sharing and home cooking alongside improved attention to diet (FSA & Ipsos Mori, 2020). It is possible that a shift towards home cooking, if it continues, could ultimately lead to reductions in chronic diet‐associated diseases, like hypertension, obesity, diabetes and cardiovascular disease (Oaklander, 2020).

Although parent’s gender was found non‐significant predictor of familial healthy eating behavior, mothers in our sample were more likely to encounter negative emotions during the pandemic, which adversely affected the healthiness of their familial eating behavior. Moreover, parent’s gender was found to interact with COVID‐19 perceptions effects on familial healthy eating behavior, where fathers were more likely to engage in such behavior in response to COVID‐19 perceptions than mothers. These results substantiate the findings of other relevant studies. Women have been reported to experience greater anxiety, sadness and other negative emotions following stress than men (e.g., Ahnlund & Frodi, 1996; Brody, Hall & Stokes, 2018; Chaplin *et al*., 2008; Derdikman‐Eiron, Indredavik, Bratberg, Taraldsen, Bakken & Colton, 2011; Fehm *et al*., 2005; NHS, 2016; Reichenberger, Pfaller, Forster, Gerczuk, Shiban & Mühlberger, 2019) which could make women more vulnerable to stress‐related weight gain than men (Cleveland Clinic, 2019). Also, in a family setting, mothers tend to experience higher levels of stress about parenting (Scher & Sharabany, 2005). Previous research has found that having a child with health conditions can affect how mothers feed their children. For instance, compared to fathers, mothers might engage more in unfavorable feeding practices if the child was food‐allergen (Mahmoud & Grigoriou, 2019; Mahmoud, Hack‐Polay, Fuxman, Naquiallah *et al*., 2020).

### Practical implications

The research has several significant implications for practitioners and policymakers. The first of these implications is to consider the gender differences noted and develop targeted counseling and training programs to raise awareness of the impact of the COVID‐19 era constraints and their impacts on food choice and, by ricochet, on health. It is clear that there are persisting gender differences in the household. Providing such support could generate enhanced psychological capital in parents of different gender, which they could transmit to their children. As suggested in the literature, parental influence on children’s health and their future food and lifestyle choices cannot be underestimated (Diener *et al*., 2010; Mahmoud, Hack‐Polay, Fuxman, Naquiallah *et al*., 2020; Steptoe *et al*., 1995).

There is a role for government, schools, the workplace, etc. in developing and promoting a campaign of healthy eating during the pandemic as there is significant evidence connecting healthy eating habits with an individual’s ability to cope with illness. We propose intervention with children specifically in recognition that teenagers could develop awareness and good food habits through school programmes and bring these to the families to help sensitize parents. Our perspective is to address the consequences of the covid‐19 disease (e.g., unhealthy food habits, lack of exercises, unhealthy weight gains, etc.) as vigorously as the causes of contamination lauded in the campaign advocating preventive measures with the slogan “wash your hands – do not touch your face – avoid crowded places.” Failure at taking such firm actions could leave society with a significant health problem in the post‐pandemic era.

### Research implications and limitations

Although this inquiry was carried out in the United Kingdom, the results should have adequate external validity in other Western nations and territories. Furthermore, given the globalization of the current pandemic crisis, our results’ generalizability can extend to other contexts. Still, further replications should be performed to validate our hypothetical model either in developed, developing or under‐developed contexts. Such replications in a cross‐cultural context would offer a clearer view of how parents’ COVID‐19 perceptions effects on familial eating habits might vary across social classes, ethnic groups or cultures. Additionally, due to challenges raised by the movement restrictions to the surveying team to adopt a probability sampling approach, one limitation of this study is that we drew our results upon a non‐probability sample.

In comparison, non‐probability sampling could establish a threat to external validity and, thus, the generalizability of research results. Notwithstanding, previous research has recommended using a non‐probability sample for empirical investigations in extreme contexts, for example, pandemic (Mahmoud, Grigoriou, Fuxman & Reisel, 2020). However, we still encourage subsequent research to employ non‐probability sampling approaches, if possible, although many risks could accompany the data collection process amidst the new waves’ lockdowns.

Much criticism has hailed on the use of the cross‐sectional design in determining causal links in structural models. Nonetheless, cross‐sectional study conclusions can still be deemed valid and interpretable as long as, according to Tharenou, Donohue and Cooper (2007), they are based on sound theoretical premises. Also, longitudinal design to reflect causality has been exaggerated that it only presents limited benefits over the cross‐sectional design in most instances in which it is employed (Spector, 2019). Despite our justification, future research where longitudinal data collection would be possible is encouraged.

Finally, it is unfortunate that our research did not include parent’s or child’s food allergy in the hypothesized model despite the critical role that has been assigned to it in previous investigations on parental food choices (e.g., Fiocchi *et al*., 2010; Mahmoud & Grigoriou, 2019; Mahmoud, Hack‐Polay, Fuxman, Naquiallah *et al*., 2020; Wu, Franciosi, Rothenberg & Hommel, 2012). The tiny number of our sample participants who were (or their children) with food allergy did not allow us to test the possible variance that could occur to the hypothesized model. Therefore, we highly endorse future research that would employ sampling approaches (e.g., quota sampling) to substantiate allergens’ representation in studies of familial eating behavior.

## Data Availability

Research data are not shared.

## Appendix

**Table A1:** Multi‐item measures utilized in the current study

Variable	Item	Assessment	Source
COVID‐19 perceptions	I believe that the effect the coronavirus pandemic has had on people is	5‐point Likert scale. 5‐point Likert scale. 1 = positive, 5 = negative 5‐point Likert scale. 1 = "strongly disagree," 5 = "strongly agree"	(Current study; Mahmoud, Grigoriou, Fuxman, Reisel, *et al.*, 2020)
	The coronavirus pandemic is making me feel discomfort		
	I feel worried about what could happen if any of my family or friends caught the virus		
Family healthy eating behaviors	High in fibre and roughage	5‐point Likert scale. 1 = "Very rarely or never," 5 = "Very often or always"	(Steptoe *et al*., 1995)
	Nutritious		
	Contains lots of vitamins and minerals		
	High in protein		
	Keeps family members healthy		
	Good for family members’ skin/teeth/hair/nails etc		
Negative emotions/experience	Afraid	5‐point Likert scale. 1 = "Very rarely or never," 5 = "Very often or always"	(Diener *et al*., 2010)
	Angry		
	Bad		
	Negative		
	Sad		
	Unpleasant		

**Table A2:** Descriptive statistics

Construct	Full sample: *N* = 384		Fathers: *N* = 84		Mothers: *N* = 300	
	Mean	STDEV	Mean	STDEV	Mean	STDEV
COVID‐19 perceptions	3.7257	0.94771	3.0952	0.49784	3.9022	0.96889
Negative emotions	2.8368	1.00332	1.9762	0.67323	3.0778	0.94727
Healthy eating	3.6059	0.67837	3.6905	0.22723	3.5822	0.75667
